# Polymorphism rs143384 *GDF5* reduces the risk of knee osteoarthritis development in obese individuals and increases the disease risk in non-obese population

**DOI:** 10.1186/s42836-023-00229-9

**Published:** 2024-03-01

**Authors:** Vitaly Novakov, Olga Novakova, Maria Churnosova, Inna Aristova, Marina Ponomarenko, Yuliya Reshetnikova, Vladimir Churnosov, Inna Sorokina, Irina Ponomarenko, Olga Efremova, Valentina Orlova, Irina Batlutskaya, Alexey Polonikov, Evgeny Reshetnikov, Mikhail Churnosov

**Affiliations:** 1 Department of Medical Biological Disciplines, Belgorod State National Research University, Belgorod, 308015 Russia; 2 Department of Biology, Medical Genetics and Ecology and Research Institute for Genetic and Molecular Epidemiology, Kursk State Medical University, Kursk, 305041 Russia

**Keywords:** Candidate genes, Knee osteoarthritis, Obesity, SNP, *GDF5*, Association

## Abstract

**Background:**

We investigated the effect of obesity on the association of genome-wide associative studies (GWAS)-significant genes with the risk of knee osteoarthritis (KOA).

**Methods:**

All study participants (*n* = 1,100) were divided into 2 groups in terms of body mass index (BMI): BMI ≥ 30 (255 KOA patients and 167 controls) and BMI < 30 (245 KOA and 433 controls). The eight GWAS-significant KOA single nucleotide polymorphisms (SNP) of six candidate genes, such as *LYPLAL1* (rs2820436, rs2820443), *SBNO1* (rs1060105, rs56116847), *WWP2* (rs34195470), *NFAT5* (rs6499244), *TGFA* (rs3771501), *GDF5* (rs143384), were genotyped. Logistic regression analysis (gPLINK online program) was used for SNPs associations study with the risk of developing KOA into 2 groups (BMI ≥ 30 and BMI < 30) separately. The functional effects of KOA risk loci were evaluated using in silico bioinformatic analysis.

**Results:**

Multidirectional relationships of the rs143384 *GDF5* with KOA in BMI-different groups were found: This SNP was KOA protective locus among individuals with BMI ≥ 30 (OR 0.41 [95%CI 0.20–0.94] recessive model) and was disorder risk locus among individuals with BMI < 30 (OR 1.32 [95%CI 1.05–1.65] allele model, OR 1.44 [95%CI 1.10–1.86] additive model, OR 1.67 [95%CI 1.10–2.52] dominant model). Polymorphism rs143384 *GDF5* manifested its regulatory effects in relation to nine genes (*GDF5, CPNE1, EDEM2, ERGIC3, GDF5OS, PROCR, RBM39, RPL36P4, UQCC1*) in adipose tissue, which were involved in the regulation of pathways of apoptosis of striated muscle cells.

**Conclusions:**

In summary, the effect of obesity on the association of the rs143384 *GDF5* with KOA was shown: the “protective” value of this polymorphism in the BMI ≥ 30 group and the “risk” meaning in BMI < 30 cohort.

**Supplementary Information:**

The online version contains supplementary material available at 10.1186/s42836-023-00229-9.

## Introduction

Osteoarthritis (OA) is a whole-joint disease involving all joint tissues (cartilage, subchondral bone, synovial membrane, meniscus, and infrapatellar fat pad) [1]. Knee osteoarthritis (KOA) represents the most common joint disease with a systemic metabolic component [2]. KOA affects 16% of the population over the age of 15 worldwide, and in 2020 about 654.1 million people over the age of 40 suffered from this condition [3]. The prevalence of KOA is constantly on the rise, primarily due to the increasing average life expectancy, as well as higher rates of obesity among the population [4]. Across the globe, KOA is considered to be a significant public health problem that has serious social and economic consequences [5]. The main reasons for KOA patients to seek medical help are pain and loss of joint function [6]. Total knee replacement is currently the most used treatment option for end-stage KOA [7]. The total number of knee replacements is expected to grow to 3.48 million by 2030 [8]. The material costs associated with KOA account for about 0.5% of gross domestic product in developed countries [9].

A number of factors, such as age, female sex, obesity, genetics, joint injuries, vitamin D deficiency, etc., have been identified to be the leading risk factors for KOA [10, 11]. Among them, obesity and overweight are two major modifiable risk factors [12]. Traditionally, one of the KOA causes is believed to be the over-weight-related biomechanical load on the joint [13]. At the same time, it is known that adipokines and proinflammatory cytokines produced by systemic and local adipose tissues are involved in cartilage degradation, synovial membrane inflammation, and bone erosion [2]. In addition, adipose tissue in the knee joint (the infrapatellar and suprapatellar fat pads, and other small fat pads such as posterior knee fat pad and posterior suprapatellar fat pad [prefemoral]) can interact with neighboring tissues, thereby potentially affecting homeostasis of joint and leading to destructive processes in KOA due to pro-inflammatory mediators [2, 14, 15]. Therefore, KOA is presently considered to be a disease entity and is aggravated by a metabolic component associated with adipose tissue [13]. Obese or overweight people are three times more likely to develop KOA than individuals with normal body weight [16]. It is known that the progression of the disease is more often observed in obese/overweight KOA patients [17]. Obviously, BMI plays a substantial role in the predisposition to KOA, but the mechanisms (including genetic one) underlying this relationship remain unclear.

The KOA is a polygenic disease [18, 19]. Thanks to genome-wide association studies (GWAS), to date, more than 80 polymorphic loci associated with the development of KOA are known [20]. Several studies have examined the relationship of various genetic variants with BMI in KOA patients [21–28]. At the same time, despite numerous data indicating a significant relationship between BMI and the development/progression of KOA [12, 13, 17, 29], genetic studies revealing the role of individual GWAS-significant polymorphic loci in the disease formation in interaction with BMI are very limited [25, 28].

Therefore, in this study, we investigated the possible effect of obesity on the GWAS-significant genetic association with the risk of developing KOA.

## Materials and methods

### KOA patients and controls

The study was of “patient-control” design, and involved 1,100 subjects (500 patients with KOA and 600 individuals without KOA), who were divided into two groups in terms of BMI: group I, including individuals with a BMI ≥ 30 (255 KOA patients and 167 controls); and group II, consisting of subjects with a BMI < 30 (245 KOA patients and 433 controls). The anthropometric indicators (weight, height) were gathered by previously outlined standard methods [30]. BMI was computed by using the standard method (ratio between body weight (in kilograms) and height (in meters) in squared [kg/m^2^]) [31]. We used well-accepted BMI grading, i.e., < 18.5 (underweight), 18.5–24.9 (normal weight), 25.0–29.9 (overweight), and ≥ 30 (obese) [32]. The KOA patients for the study were selected by certified orthopedic-traumatologists over the period from February 2016 to December 2018, based on “Belgorod City Hospital No. 2” (Department of Orthopedics and Traumatology). The study was approved by the ethics committee of this hospital.

Several inclusion criteria were used in the formation of KOA and control cohorts:

- (1) individuals of European origin, who were born and living in the Central region of Russia and were not related to each other [33–35]; (2) aged 40 years or older; (3) availability of informed consent to take part in the study; (4) the KOA group included patients with [36], (i) primary KOA of the knee joint, diagnosed against the American College of Rheumatology [37], (ii) KOA radiological stage by J. Kellgren-J. Lawrence (K/L) ≥ 2 [38], (iii) the presence of pain syndrome more than 40 points on the Visual Analog Scale (VAS) [39]; (5) the control group included subjects who did not have any pathology of the musculoskeletal system. Exclusion criteria were as follows: (1) the presence of severe hypertension, coronary heart disease, diabetes mellitus, renal-hepatic insufficiency, oncological diseases, systemic connective tissue diseases, joint injuries in the anamnesis, inflammatory joint diseases, congenital malformations of the musculoskeletal system, (2) refusal to participate in the study.

### SNP selection criteria and genotyping

The eight GWAS-significant KOA SNPs of six candidate genes, such as *LYPLAL1*—*lysophospholipase like 1* (rs2820436, rs2820443), *SBNO1*—*strawberry notch homolog 1* (rs1060105, rs56116847), *WWP2*—*WW domain containing E3 ubiquitin protein ligase 2* (rs34195470), *NFAT5*—*nuclear factor of activated T cells 5* (rs6499244), *TGFA*—*transforming growth factor alpha* (rs3771501), *GDF5*—*growth differentiation factor 5* (rs143384), were selected for the genetic study based on previously registered GWAS associations (*P* ≤ 5 × 10^–8^) of these loci with KOA in European populations [40–44] (Table S1, Supplementary Information) and existence of the functional value [45–47]. To determine the loci functionality, the HaploReg database was used [48] (Table S2, Supplementary Information).

Genomic DNA of participants was isolated from peripheral blood (The buffy coat containing leucocytes was used.) by using a well-established phenol–chloroform-ethanol extraction/concentration method based on a previously published laboratory protocol [49]. The purity and concentration of the isolated DNA samples were measured on a NanoDrop spectrophotometer [50]. DNA materials of KOA patients and controls (each PCR tablet contained DNA samples of patients and controls) were genotyped by real-time PCR on the CFX96-Real-Time PCR System (Bio-Rad Laboratories, Hercules, CA, USA) [51, 52] and by using specially-developed reagent kits (TestGen, Ulyanovsk, Russia). The sequences of oligonucleotide primers and probes used in SNP genotyping are presented in Table S3 (Supplementary Information). To control the quality of experimental data, ≈7%–10% of the randomly selected DNA specimen were re-genotyped [53, 54]. A virtually complete coincidence was achieved between the repeated genotyping results with the primary data (an error of no more than 1%).

### Statistical and bioinformatic analysis

For all the considered loci in KOA patients and controls in the two study subgroups (BMI ≥ 30 and BMI < 30), we evaluated the correspondence of the observed genotype distribution to the expected one according to the Hardy–Weinberg pattern [55, 56]. The association between SNP and KOA was investigated in the two groups (BMI ≥ 30 and BMI < 30) separately by using the logistic regression (allelic/additive/dominant/recessive genetic models [57] were considered), with adjustments made for age, sex, BMI, occupation-related physical workload, hereditary burden, the presence of concomitant pathology of the cardiovascular, musculoskeletal systems, height and leisure time physical activity (Table 1). All calculations were carried out by employing the gPLINK software [58] and were subjected to calibration for multiple comparisons (a well-established permutation test was applied) [59, 60]. Finally, a *P*_perm._ ≤ 0.025 was considered to be statistically significant (Bonferroni correction was introduced for the number of groups compared (*n* = 2)—with/without obesity) [61]. For individual SNPs, statistical power was estimated by utilizing Quanto (v.1.2.4) [62].

**Table 1:** Phenotypic characteristics of the study participants

Parameters	BMI ≥ 30			BMI < 30		
	KOA patients $\overline{{\text{X}} }$ ± SD/% (*n*)	Controls $\overline{{\text{X}} }$ ± SD/% (*n*)	*P*	KOA patients $\overline{{\text{X}} }$ ± SD/% (*n*)	Controls $\overline{{\text{X}} }$ ± SD/% (*n*)	*P*
*n*	255	167	-	245	433	-
Age, years (min–max)	52.55 ± 5.48	53.54 ± 6.15	0.12	52.84 ± 5.88	52.88 ± 6.81	0.79
Men/Women	33.73 (86) / 66.27 (169)	38.92 (65) / 61.08 (102)	0.33	49.80 (122) / 50.20 (123)	40.65 (176) / 59.35 (257)	**0.03**
BMI, kg/m^2^	34.50 ± 3.50	32.72 ± 2.32	**0.0001**	26.33 ± 2.32	25.00 ± 2.15	** < 1 × 10**^**–6**^
Height, cm	167.89 ± 7.23	166.78 ± 7.67	0.20	170.57 ± 8.23	169.23 ± 7.56	**0.02**
Alcohol (yes)	76.08 (194)	76.05 (127)	1.00	77.14 (189)	75.06 (325)	0.61
Smoker (yes)	25.10 (64)	22.16 (37)	0.56	24.49 (60)	21.48 (93)	0.42
Hereditary burden (yes)^a^	36.86 (94)	5.99 (10)	**0.0005**	41.22 (101)	15.94 (69)	**0.0005**
Occupation-related physical workload						
Low	16.08 (41)	38.92 (65)	**0.0005**	20.82 (51)	39.03 (169)	**0.0005**
Medium	53.33 (136)	44.91 (75)	0.11	46.94 (115)	45.27 (196)	0.73
High	30.59 (78)	16.17 (27)	**0.002**	32.24 (79)	15.70 (68)	**0.0005**
Leisure time physical activity						
Little	68.63 (175)	59.28 (99)	0.06	70.61 (173)	56.58 (245)	**0.001**
Irregular	24.71 (63)	28.74 (48)	0.42	25.31 (62)	31.18 (135)	0.13
Regular	6.66 (17)	11.98 (20)	0.09	4.08 (10)	12.24 (53)	**0.002**
Concomitant pathology, % (n)						
Digestive system	11.37 (29)	5.39 (9)	0.05	12.65 (31)	10.62 (46)	0.50
Cardiovascular system	35.29 (90)	14.97 (25)	**0.0005**	38.37 (94)	19.17 (83)	**0.0005**
Senitourinary system	4.71 (12)	9.58 (16)	0.08	6.94 (17)	4.39 (19)	0.21
Central nervous system	12.16 (31)	10.18 (17)	0.64	8.57 (21)	8.08 (35)	0.94
Musculoskeletal system	10.20 (26)	0 (0)	**0.0006**	5.31 (13)	0 (0)	**0.0005**
Endocrine system	11.37 (29)	8.98 (15)	0.53	8.98 (22)	5.54 (24)	0.12
Respiratory system	11.76 (30)	8.98 (15)	0.46	11.84 (29)	9.70 (42)	0.46

^a^The presence of KOA in relatives of the first degree of kinship (mother, father)

*P* values < 0.05 are shown in bold

Functionality of KOA-associated loci (epigenetic; eQTL; sQTL; protein structure change (amino acid substitution) [63]) and SNPs strongly linked with them (parameter r^2^ ≥ 0.80 [64]) were estimated by using modern bioinformatic online resources (in silico procedures) [65–67]: (a) Blood eQTL browser [68], (b) PolyPhen-2 [69], (c) GeneMANIA [70], (d) HaploReg [48], (e) GTExproject [71], (f) SIFT [72].

## Results

The main phenotypic parameters of KOA patients and KOA-free individuals in the two groups, grouped in terms of the presence/absence of obesity (BMI ≥ 30 and BMI < 30) are given in Table 1. It was found that the KOA patients with BMI ≥ 30, as well as those with BMI < 30, compared with their corresponding controls, had significantly higher BMI (*P* = 0.0001 and *P* < 1 × 10^–6^, respectively), hereditary burden (*P* = 0.0005 and *P* = 0.0005), incidences of cardiovascular (*P* = 0.0005 and *P* = 0.0005) and musculoskeletal diseases (*P* = 0.0006 and *P* = 0.0005) diseases. Among the KOA subjects (BMI ≥ 30 and BMI < 30), in comparison with their respective controls, the percentage of individuals with a high level of professional physical activity was significantly higher (1.89 times, *P* = 0.002, and 2.05 times, *P* = 0.0005) and the proportion of individuals with a low level of professional physical activity was significantly lower (2.42 times, *P* = 0.0005, and 1.87 times, *P* = 0.0005, respectively). Additionally, in KOA patients without obesity (BMI < 30), the percentage of individuals with low physical activity in their free time was significantly higher (1.25 times, *P* = 0.001) and the proportion of individuals with regular physical activity was significantly lower (3 times, *P* = 0.002), compared to the controls (Table 1). The above-mentioned environmental KOA risk/protective factors were included in the association analysis as covariates.

The statistical materials in Table S4 (Supplementary Information) (BMI < 30 cohort) and Table S5 (Supplementary Information) (BMI ≥ 30 subject) demonstrate that the distribution (observed/expected) of the studied SNPs followed the HWE law (the Bonferroni correction based on the number of examined loci was used (*P*_bonf._ = 0.00625 [0.05/8]).

Multidirectional relationships of the rs143384 *GDF5* with KOA in BMI-different groups were found: allele G of this SNP was a KOA protective genetic variant in individuals with BMI ≥ 30 (OR 0.41 [95%CI 0.20–0.94], *P* = 0.019, *P*_perm._ = 0.020, power 87.23%, recessive model) and was a disease risk variant in subjects with BMI < 30 (OR 1.32 [95%CI 1.05–1.65], *P* = 0.016, *P*_perm._ = 0.018, allele model; OR 1.44 [95%CI 1.10–1.86], *P* = 0.007, *P*_perm._ = 0.009, power 89.33%, additive model; OR 1.67 [95%CI 1.10–2.52], *P* = 0.015, *P*_perm._ = 0.012, power 81.01%, dominant model) (Table 2).

**Table 2:** Associations of the studied gene polymorphisms with KOA in subjects with BMI < 30 and BMI ≥ 30

Chr	SNP	Gene	Minor allele	*n*	Allelic model				Additive model				Dominant model				Recessive model			
					OR	95%CI		*P*	OR	95%CI		*P*	OR	95%CI		*P*	OR	95%CI		*P*
						L95	U95			L95	U95			L95	U95			L95	U95	
Individuals with BMI < 30																				
1	rs2820436	*LYPLAL1*	A	676	0.81	0.63	1.03	0.084	0.80	0.59	1.07	0.132	0.82	0.56	1.19	0.295	0.56	0.28	1.15	0.116
1	rs2820443	*LYPLAL1*	C	664	0.99	0.77	1.28	0.937	0.97	0.72	1.31	0.844	0.93	0.63	1.37	0.710	1.09	0.53	2.26	0.814
2	rs3771501	*TGFA*	A	676	1.08	0.86	1.35	0.501	1.03	0.81	1.36	0.713	1.14	0.77	1.70	0.508	0.97	0.61	1.56	0.903
12	rs1060105	*SBNO1*	T	678	1.04	0.80	1.36	0.752	1.08	0.80	1.47	0.616	0.96	0.65	1.42	0.848	1.85	0.88	3.88	0.103
12	rs56116847	*SBNO1*	A	676	0.92	0.73	1.16	0.477	0.85	0.65	1.13	0.271	0.93	0.64	1.36	0.705	0.59	0.32	1.08	0.090
16	rs6499244	*NFAT5*	A	677	1.03	0.82	1.28	0.808	1.04	0.80	1.35	0.793	1.15	0.76	1.74	0.508	0.94	0.60	1.47	0.787
16	rs34195470	*WWP2*	A	674	1.07	0.85	1.33	0.562	1.15	0.88	1.51	0.308	1.16	0.75	1.78	0.505	1.26	0.80	1.98	0.314
20	rs143384	*GDF5*	G	676	**1.32**	**1.05**	**1.65**	**0.016**	**1.44**	**1.10**	**1.86**	**0.007**	**1.67**	**1.10**	**2.52**	**0.015**	1.61	1.02	2.54	0.043
Individuals with BMI ≥ 30																				
1	rs2820436	*LYPLAL1*	A	422	0.72	0.48	1.07	0.101	0.68	0.43	1.06	0.090	0.63	0.34	1.17	0.140	0.54	0.21	1.38	0.195
1	rs2820443	*LYPLAL1*	C	420	0.89	0.59	1.36	0.600	0.88	0.56	1.38	0.570	1.00	0.54	1.84	1.000	0.54	0.20	1.40	0.202
2	rs3771501	*TGFA*	A	421	0.87	0.59	1.27	0.467	0.85	0.56	1.30	0.448	0.74	0.38	1.45	0.384	0.88	0.42	1.85	0.730
12	rs1060105	*SBNO1*	T	422	1.25	0.76	2.03	0.378	1.47	0.85	2.52	0.168	1.65	0.86	3.15	0.130	1.29	0.31	5.40	0.724
12	rs56116847	*SBNO1*	A	422	0.86	0.58	1.27	0.444	0.84	0.55	1.31	0.451	0.60	0.32	1.13	0.115	1.42	0.56	3.57	0.459
16	rs6499244	*NFAT5*	A	422	1.27	0.86	1.87	0.234	1.42	0.92	2.21	0.114	1.65	0.86	3.13	0.130	1.53	0.69	3.42	0.296
16	rs34195470	*WWP2*	A	422	1.06	0.72	1.56	0.762	1.05	0.67	1.64	0.835	0.96	0.48	1.91	0.913	1.21	0.55	2.67	0.631
20	rs143384	*GDF5*	G	422	0.85	0.58	1.25	0.415	0.78	0.50	1.21	0.263	1.03	0.54	1.95	0.940	**0.41**	**0.20**	**0.94**	**0.019**

All results were obtained after adjustment for covariates

*OR* Odds ratio, *95%CI* 95% confidence interval

*P* values < 0.05 are shown in bold

### Functionality of KOA-associated rs143384 GDF5 (in silico data)

The polymorphism rs143384 (located in the 5’-UTR region of the *GDF5* gene) and 9 SNPs strongly linked to it exhibited various epigenetic effects (They are significant for the chromatin structure in the regions of potential promoters and enhancers, and affect the interaction of DNA with many transcription factors such as Ascl2, Foxa, TFE, Ets, Pitx2, SP2, LUN-1, EBF, Mxi1, Myf, Myc, NRSF, TAL1, YY1, Zfx, E2A, ELF1, etc.) (Table 3), including cell cultures of adipose (adipose derived mesenchymal stem cells, epigenomeID-E025/mesenchymal stem cells derived adipocyte cultured cells, epigenomeID-E023/nuclei of adipose, epigenome ID-E063) (Data were obtained from the Haploreg database [48]).

**Table 3:** Regulatory effects of the KOA-associated polymorphism rs143384 and SNPs in high LD (r^2^ ≥ 0.80) (data of the Haploreg database)

Chr	Position (hg38)	LD		variant	Ref	Alt	EUR frequency	Conserved sequence (SiPhy algoritm)	Promoter histone marks	Enhancer histone marks	DNase hypersensitive site	Proteins bound	Motifs changed	NHGRI/EBI GWAS hits	GRASP QTL hits	Selected eQTL hits	GENCODE genes	dbSNP Base (functional annotation)
		r^2^	D															
20	35414469	0.8	-0.94	rs34091597	CAT	C	0.61						Foxa, TFE			24 hits	2.3 kb 5’ of UQCC	
20	35417411	0.81	-0.95	rs6060401	C	T	0.62						Ets, Pitx2			55 hits	5.3 kb 5’ of UQCC	
20	35417437	0.81	-0.95	rs6060402	T	C	0.62						SP2			64 hits	5.3 kb 5’ of UQCC	
20	35419167	0.8	-0.94	rs6141551	T	C	0.61						LUN-1			54 hits	7 kb 5’ of UQCC	
20	35420334	0.81	-0.95	rs7262358	C	T	0.62						4 altered motifs			57 hits	8.2 kb 5’ of UQCC	
20	35420820	0.81	-0.95	rs6142381	G	A	0.62						9 altered motifs			55 hits	8.7 kb 5’ of UQCC	
20	35431781	0.84	0.98	rs224329	C	T	0.38		LIV	5 tissues	4 tissues		EBF	1 hit	1 hit	63 hits	1.2 kb 5’ of GDF5OS	
20	35436182	0.83	0.99	rs224333	G	A	0.37	+	7 tissues	6 tissues	ESDR		4 altered motifs	1 hit		57 hits	GDF5	intronic
20	35437976	1	1	rs143384	A	G	0.41	+	9 tissues	13 tissues	16 tissues		Ascl2	3 hits		47 hits	GDF5	5’-UTR
		0.85	0.99	rs78110303	A	G	0.37	+	9 tissues	14 tissues	31 tissues		4 altered motifs			24 hits	GDF5	5’-UTR

*Chr* Chromosome, *LD* linkage disequilibrium, *r*^2^ Pearson correlation coefficient, *D* linkage disequilibrium coefficient, *Ref* reference allele, *Alt* alternative allele

The Blood eQTL browser showed that the minor allele G rs143384 is associated (*P*_FDR_ = 0) with reduced mRNA level of *UQCC* (Z parameter -6.35) and *CEP250* (-5.74) genes and a high production of *EIF6* mRNA (11.29) in peripheral blood (Table S6, Supplementary Information). In addition, the involvement of the three loci (rs6060402, rs224329, rs224333) highly coupled with rs143384 in transcriptional regulation of the above three genes in peripheral blood was displayed in Table S7 (Supplementary Information).

Based on experimental data of GTEx portal, rs143384 *GDF5* has been identified as a modulator of multiple genes expression (21 genes/more 30 organs) and alternative splicing (8 genes/above 20 organs), including eight genes in adipose tissue (expression quantitative locus/trait [eQTL]: *CPNE1, EDEM2, GDF5, PROCR, RPL36P4, UQCC1*; splicing quantitative locus/trait [sQTL]: *RBM39, UQCC1, ERGIC3*) (Tables S8 and S9, Supplementary Information). Remarkably, the G allele of the rs143384 locus was correlated with low expression/splicing of four/two genes (*CPNE1, EDEM2, PROCR, UQCC1/ERGIC3, RBM39*) in adipose tissue and high expression/splicing of two/one genes (*GDF5, RPL36P4/UQCC1*) in this tissue (Tables S8 and S9, Supplementary Information). Among nine high-linked SNPs, eight loci were eQTL (21 genes including seven genes in adipose tissue: *CEP250, CPNE1, EDEM2, PROCR, RP4-614O4.13, RPL36P4, UQCC1*) (Table S10, Supplementary Information) and sQTL (10 genes including five genes in adipose tissue: *EIF6, ERGIC3, FER1L4, RBM39, UQCC1*) (Table S11, Supplementary Information).

**Fig. 1:**
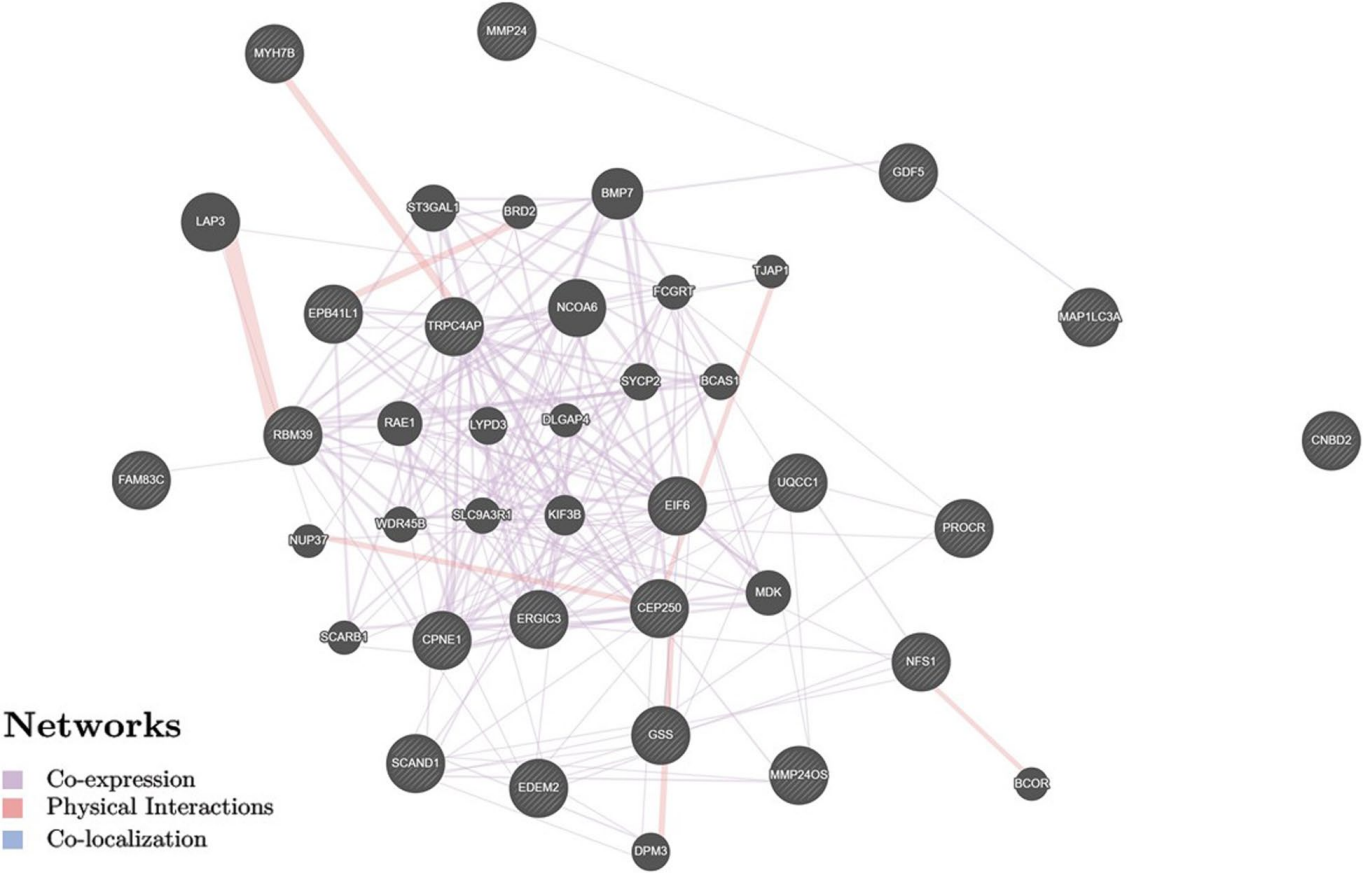
The interaction networks of the candidate genes associated with rs143384 (eQTL/sQTL/regulatory effects this SNP) inferred using GeneMANIA (http://genemania.org)

**Fig. 2:**
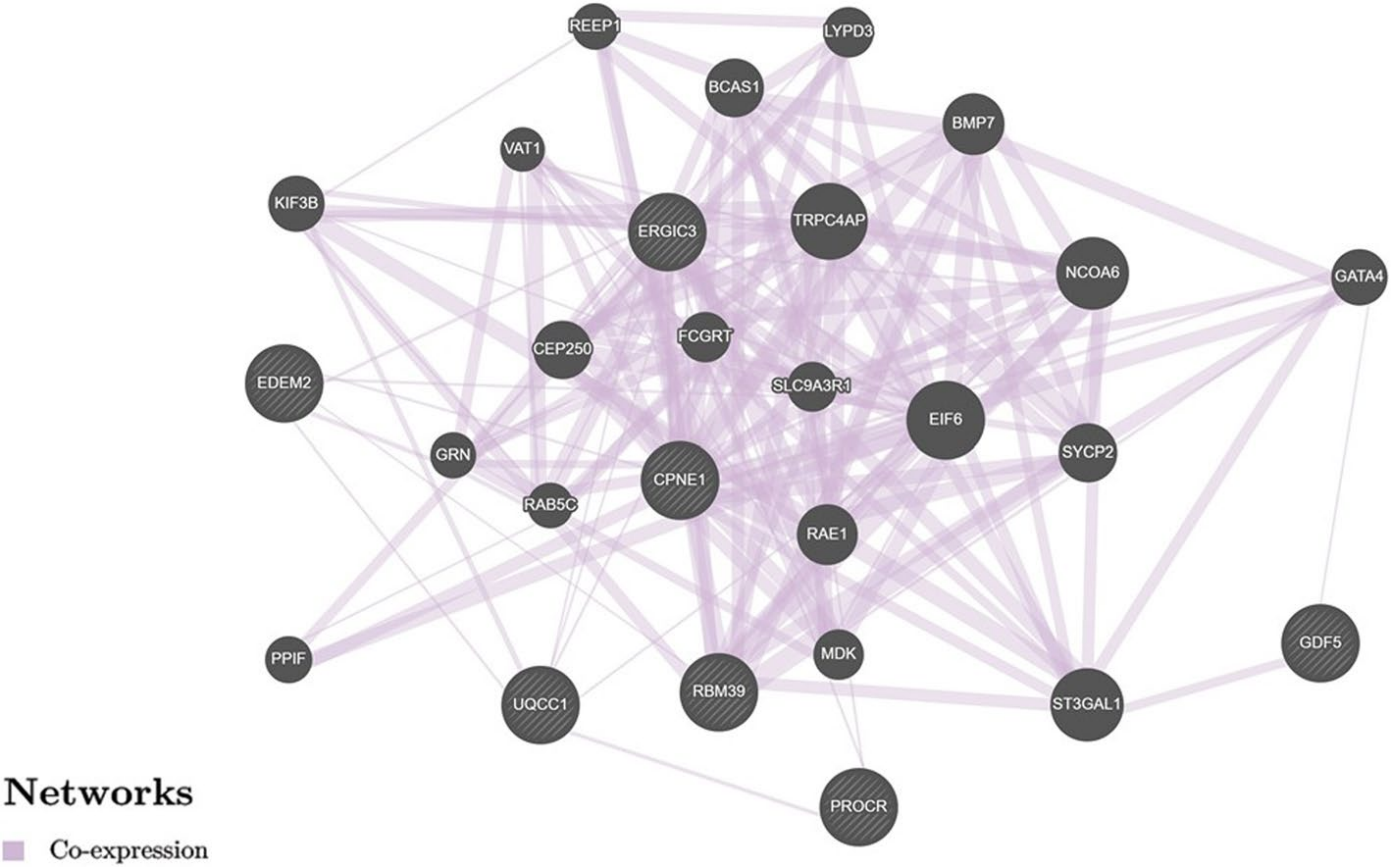
The interaction networks of the candidate genes in adipose tissue associated with rs143384 (eQTL/sQTL/regulatory effects this SNP) inferred using GeneMANIA (http://genemania.org)

Overall, first of all, we found very pronounced rs143384 *GDF5* functionality in relation to 26 genes in a variety of organs (more than thirty ones) which interactions due co-expression (the percentage contribution was the highest and amounted to 85.77%), physical interactions (12.16%) and co-localization (2.07%) (Fig. 1, GeneMANIA data) with the leading role of paired interactions such as *LAP3–RBM39, NQO2–NQO1, TRPC4AP–MYH7B, BRD2–EPB41L1, DPM3–CEP250* (weight indicators 0.21–0.62) (Table S12, Supplementary Information). Secondly, considerable functionality of the rs143384 *GDF5* in adipose tissue in relation to nine genes (*CPNE1, EDEM2, ERGIC3, GDF5, GDF5OS, PROCR, RBM39, RPL36P4, UQCC1*) was found with complete dominance (100%) of co-expression in their interactions (Fig. 2 and Table S13, GeneMANIA data) and involved above genes set in regulation of the pathways of apoptosis of striated muscle cells (*P*_FDR_ = 0.004).

## Discussion

In the present study, the effect of obesity on the association of the rs143384 *GDF5* with KOA was shown: allele G of this SNP was a KOA protective factor in individuals with BMI ≥ 30 (OR 0.41) and disease risk marker in individuals with BMI < 30 (OR 1.32–1.67). Polymorphism rs143384 *GDF5* exerted its regulatory effects in relation to 9 genes in adipose tissue.

A multitude of literature data confirmed that high BMI and obesity are the leading risk factors for the development and progression of OA [29, 73, 74]. In the sample we studied, obesity was also a significant risk factor for KOA (OR = 6.73, *P* = 0.005). It is known that the key points in the pathogenesis of KOA in obesity are determined by excessive mechanical stress on the joint, chronic inflammation in adipose tissue and dyslipidemia, secretion of proinflammatory cytokines and adipokines by adipose tissue; cytokine secretion by infrapatellar adipose tissue [2]. Adipokines (leptin, resistin, etc.) and cytokines (TNFA, IL1, IL6) produced by adipose tissue (both local and systemic adipose tissue), in turn, can affect pathological processes in the tissues of the joint and bones, such as cartilage degradation, inflammatory processes in the synovial membrane, bone erosion [2, 75, 76]. It is worth noting that infrapatellar adipose tissue or Goff’s fat cushion plays important roles in the pathogenesis of KOA [2]. On the one hand, the damping role of this adipose tissue is known to be due to the damping of mechanical stress under load on the joint (A redistribution of adipose tissue takes place.) [77]. On the other hand, KOA is often accompanied by inflammation of infrapatellar adipose tissue with increased expression of inflammatory mediators such as IL-6, adipsin, visfatin and adiponectin, which also support inflammatory processes in other joint tissues [78, 79]. It has been shown that an increase in body mass index by 5 kg/m^2^ is associated with a rise of 35% in the risk of developing KOA [80]. It is known that obesity often leads to the progression of KOA [17] and can cause a more severe course of the disease [81]. A study by Vasilic-Brasnjevic et al. showed that obesity (BMI ≥ 30 kg/m^2^) was a risk factor for the development of stage 3–4 KOA in patients older than 50 years [81]. The presence of overweight, grade I and II obesity increased the risk of KOA by 2, 3.1 times and 4.7 times, respectively [73]. Takahashi et al. [17] demonstrated that 75% of obese KOA patients had disease progression (assessed on the Kellgren-Lawrence scale).

OA and obesity are diseases resulting from the interaction of multiple genetic and environmental factors [82–85] and sharing common pathophysiological mechanisms [29]. Obesity is considered a chronic inflammatory disease characterized by the production of cytokines and cytokine-like molecules (adipokines) that can affect various body tissues [86], including knee joint tissues. It is worth noting that the inflammatory reaction is also one of the main pathogenetic links of OA [2, 87]. The relationship between different genetic variants and BMI in KOA patients has been shown [21–28], among which there are a large number of genes associated with metabolic disorders (*FTO, ADIPOQ, LEP, SREBP2*) and other genes (*GDF5, TGFB1,* etc.). At the same time, the effect of overweight and obesity on the association of GWAS-significant loci (rs8044769 *FTO* and rs143383 *GDF5*) with KOA has been examined only in a small number of studies [25]. It should be noted that no significant association was found between the GWAS locus rs8044769 *FTO* and KOA in overweight and obese patients [25]. Conversely, Zhang et al. showed that the rs143383 *GDF5* was associated with KOA both in subjects with BMI ≥ 24 (OR = 2.36–2.45) and in those with BMI < 24 (OR = 1.63–3.77). Thus, the allele T of the rs143383 *GDF5* was a risk factor for KOA in both groups [28]. According to our data, the variant G rs143384 *GDF5* was a KOA risk factor in individuals with BMI < 30 (OR = 1.32–1.67) and a protective factor against KOA in BMI ≥ 30 subjects (OR = 0.41). The association of *GDF5* gene (rs143384) with KOA was established in four previously published GWAS [40, 42–44]. Two papers [40, 43] reported the association of the allelic variant A (rs143384) with KOA in Europeans (parameter OR = 1.10 was the same in both studies), and one study [44] showed the association in mixed samples of European and Asian origins (OR = 1.07). It is worth noting that, in these three GWAS, the allele A of the *GDF5* locus (rs143384) is risky for the development of KOA. Another study [42] demonstrated that the allele G of rs143384 was a KOA protective factor in Caucasians (OR = 0.91). It should be noted that, in our work, the allele G of rs143384 also was of protective value for KOA in the BMI ≥ 30 group.

There are a number of studies demonstrating the relationship between the rs143384 *GDF5* and various musculoskeletal pathologies of the lower extremities, including OA of other sites or body parts [88–94]. Some studies have identified associations of the rs143384 allele variant A with knee pain [90, 92, 94]. Other studies have shown the connection of this *GDF5* gene locus with hip dysplasia [89, 91], OA of the hand [93], and congenital hip dislocation [88].

The relationship between rs143384 *GDF5* and body weight, as well as various anthropometric indices (body fat distribution, waist-to-hip ratio, waist-hip index, etc.), which may be associated with overweight or obesity, was demonstrated in previously GWAS [95–101]. The G allele (rs143384) has been found to be associated with lower body fat distribution (leg fat ratio) (β = -0.031, *P* = 3 × 10^–43^) [98], waist-to-hip ratio adjusted for BMI (β = -0.035, *P* = 3 × 10^–28^) [99], waist-hip index (β = -0.031, *P* = 6 × 10^–23^) [99]; in turn, allele A (rs143384) was linked to a higher waist-to-hip ratio adjusted for BMI (β = 0.02, *P* = 2 × 10^–27^) [97]. On the contrary, other studies [95, 96] showed that the A allele of rs143384 was correlated with a lower hip circumference adjusted for BMI (β = -0.044, *P* = 1 × 10^–31^) [95], (β = -0.042, *P* = 3 × 10^–7^) [96]. Association of rs143384 *GDF5* with body weight has been shown in two papers [96; 100], in which the G allele was associated with weight gain (β = 0.028, *P* = 3 × 10^–57^) in the mixed samples of Europeans and Asians [100], and the A allele had a link with weight loss (β = -0.041, *P* = 2 × 10^–10^) in Europeans [96]. Hübel et al. revealed that rs143384 *GDF5* was associated with fat-free muscle mass (β = -0.390, *P* = 6 × 10^–68^) [101] and a study by Guilherme et al. found that the G rs143384 allele of the *GDF5* gene was associated with a low BMI in Caucasians (*P* = 1.2 × 10^–14^) [102]. Thus, it should be mentioned that, on the one hand, the association of rs143384 *GDF5* with various anthropometric characteristics was proven in several previously GWAS; on the other hand, there is inconsistencies among the results about the association of this allelic variant with the aforementioned characteristics (risk/protective effect on BMI/body fat distribution/waist-to-hip ratio of different allelic variants of rs143384) in various cohorts (populations). Our study also revealed a multidirectional nature of the association between the rs143384 *GDF5* and KOA association (the risky nature in individuals with BMI < 30 and the protective role in the group with BMI ≥ 30).

Interestingly, this study (in silico materials) demonstrated that, in adipose tissue, rs143384 *GDF5* had considerable functionality (expression; splicing; epigenetic) in relation to nine genes (*GDF5, CPNE1, EDEM2, ERGIC3, GDF5OS, PROCR, RBM39, RPL36P4, UQCC1*) involved in regulation of pathways of apoptosis of striated muscle cells. Moreover, the G allele rs143384 was associated with increased *GDF5* gene expression. Premised on this, it can be assumed that in obese individuals with the G allele, the amount of the protein product of the *GDF5* gene will be maximum (plenty of adipose tissue due to GDF5 production area and the presence of a highly productive allele G rs143384) and significantly exceed the level of *GDF5* expression (GDF5 production) in obese individuals without the G allele (a lot of adipose tissue but the presence of a low-productive allele A rs143384). This may explain the protective value for KOA of the highly productive allele G rs143384 in obese individuals, established in our study. GDF5 (growth differentiation factor 5) is a member of the bone morphogenetic protein (BMP) gene family and the TGF-beta superfamily and plays an important role in skeletal development [103], inflammatory reactions, and tissue damage [104]. Overexpression of GDF5 in human mesenchymal stem cells leads to increased chondrogenesis in vitro [105]. In mice models of OA, high expression of GDF5 in the cartilage was detected during its recovery after unilateral destabilization of the medial meniscus [106]. Allelic variants A and G rs143384 exert an important modifying effect on the KOA-risk impact of other loci. It was revealed that the T allele rs143383 (It was associated with the OA risk), which is linked to rs143384 (r^2^ = 0.82), caused the reduced transcription of the *GDF5* gene in chondrogenic cells [107–109]. It has been shown that the rs143384 locus is able to influence the “phenotypic effects” of rs143383 with respect to the *GDF5* gene (These results were obtained on the model of the luciferase reporter assays of *GDF5* promoter/5’-UTR constructs in the chondrogenic (CH8), adipogenic (SW872) and osteogenic (MG63) cell lines). The T allele of rs143383, which is risky for OA, causes a decrease in luciferase activity relative to the alternative allele C for it only in the presence of the A allele of rs143384 [110]. Increased expression of the *GDF5* gene was observed in brown adipose tissue in obese mice [111]. The study by Yang et al. showed that systemic overexpression of GDF5 in adipocytes reduced non-alcoholic liver obesity caused by a high-fat diet in mice [112]. Pei et al. exhibited that GDF5 played an adipogenic role in the differentiation of 3T3-L1 preadipocytes [113]. Thus, the *GDF5* gene is characterized by a pleotropic effect and, accordingly, affects not only KOA, but also the processes taking place in adipose tissue, which is just as important, if indirectly, for the pathophysiology of KOA. In general, as we can assume in obese individuals, the highly productive G allele rs143384 (which determines overexpression of GDF5) acts as a protective factor against KOA due to the apparent effects of high concentrations of GDF5 (increased chondrogenesis, etc.).

At the same time, we obtained data on the KOA-risk role of polymorphism rs143384 *GDF5* (allele G) in non-obesity individuals. We speculate that this relationship may be based on the following mechanisms. Firstly, a significant disadvantage of expression of GDF5 in individuals with a low content of adipose tissue (little adipose tissue due to small source of production of GDF5) and consequently weak chondrogenic and adipogenic effects of GDF5 led to an increased risk of developing KOA. Secondly, in individuals with low fat mass, an increased risk of KOA development in the presence of the G allele rs143384 may be associated with other genes whose expression/splicing level is affected by this polymorphism (*CPNE1, EDEM2, PROCR, UQCC1, RPL36P4/ERGIC3, RBM39*). For instance, due to a significant “deficiency” of protein products of genes (*CPNE1, EDEM2, PROCR, UQCC1*), their expression can be extremely reduced in individuals carrying reduced fat mass and a low-productive G allele rs143384 (KOA risk factor in individuals without obesity), which may, as an important pathogenetic factor, significantly contribute to the development of KOA. So, *CPNE1,* encoding Copine1, a soluble calcium-dependent membrane-binding protein, affects the length of myotubes (knockdown of *CPNE1* gene increases the length of myotubes) and works as a modifier of muscle mass in humans in vitro, though it is not definitively clear how alterations in myogenesis indicators in vitro relate to the hypertrophy/hyperplasia of fiber in vivo [114]. *EDEM2* encodes an ER degradation enhancer, mannosidase alpha-like 2, involved in carbohydrate metabolism (EDEM 2 identifies misfolded endoplasmic reticulum glycoproteins and targets them for destruction), and its expression in the skeletal muscle tissue of geriatric vs. young adult animals (dogs) differed significantly, depending on the diet [115]. *PROCR,* encoding the endothelial protein C receptor), is a “key” regulator of the protein C pathway mediating the interaction between coagulation and pro-inflammatory/anti-inflammatory processes in vessels [116]. *QCC1* encodes a trans-membrane protein ubiquinol-cytochrome-*c* reductase complex chaperone and is involved in the pathophysiology of OA [117]. Moreover, it is also associated with lipid metabolism (arm fat mass) [118]. However, it is important to emphasize that there is currently no definitive or flimsy evidence on this issue in the literature, and further epidemiological and experimental studies on this theme are needed.

The data obtained in the work on the genetic features of KOA in individuals with and without obesity is traumatologically and orthopedically of practical value and can help distinguish between individuals at risk for KOA development and clinically healthy population. Taking into account the presence/absence of obesity will allow for timely implementation of measures aimed at preventing the disease (for example, achieving weight loss in obese individuals with a genetic high-risk factor for KOA (allele A rs143384), etc.).

## Conclusion

This study showed that obesity exerted an effect on the associations of the rs143384 *GDF5* with the KOA risk. This polymorphism is of “protective” value in the BMI ≥ 30 subjects and a “risk” for the development of KOA in those with BMI < 30.

### Supplementary Information

**Additional file 1: Table S1.** The literature data about associations of the studied polymorphisms of the candidate genes with ОА.

**Additional file 2: Table S2.** The regulatory potential of the studied SNPs.

**Additional file 3: Table S3.** Sequence of oligonucleotide primers and probes for SNP candidate genes for KОА.

**Additional file 4: Table S4.** The allele and genotype frequencies of the studied SNPs in the KОА and control groups with BMI < 30.

**Additional file 5: Table S5.** The allele and genotype frequencies of the studied SNPs in the KОА and control groups with BMI ≥ 30.

**Additional file 6: Table S6.** Cis-eQTL values of the KOA-associated polymorphism rs143384 in blood.

**Additional file 7: Table S7.** Cis-eQTL values of the SNPs in high LD (r^2^≥0.80) with KOA-associated polymorphism rs143384 in blood.

**Additional file 8: Table S8.** eQTL values of the KOA-associated polymorphism rs143384.

**Additional file 9: Table S9.** sQTL values of the KOA-associated polymorphisms rs143384.

**Additional file 10: Table S10.** Effect of SNPs in high LD (r^2^ ≥ 0.80) with the KOA-associated polymorphisms rs143384 on gene expression level.

**Additional file 11: Table S11.** Effect of SNPs in high LD (r^2^ ≥ 0.80) with the KOA-associated polymorphisms rs143384 on alternative splicing (cis-sQTL).

**Additional file 12: Table S12.** Results of the gene-gene interaction analysis of the candidate genes associated with rs143384 (functionality in various tissue/organs.

**Additional file 13: Table S13.** Results of the gene-gene interaction analysis of the candidate genes associated with rs143384 (functionality in adipose tissue).

## Data Availability

The data generated in the present study are available from the corresponding author upon reasonable request.

## Abbreviations

BMI: Body Mass Index
GWAS: Genome-Wide Association Studies
KOA: Knee Osteoarthritis
SNP: Single Nucleotide Polymorphism
