# Supplementary material for: Polymorphism rs143384 GDF5 reduces the risk of knee osteoarthritis development in obese individuals and increases the disease risk in non-obese population
Source: Arthroplasty. 2024 Mar 1;6:12. doi: 10.1186/s42836-023-00229-9 (PMC10905832; doi:10.1186/s42836-023-00229-9)
Supplement: Supplementary file 1 — Additional file 1: Table S1. The literature data about associations of the studied polymorphisms of the candidate genes with ОА. [file 42836_2023_229_MOESM1_ESM.docx]

Supplementary table S1

The literature GWAS data about associations of the studied polymorphisms of the candidate genes with ОА

| Chr | SNP | Gene | Phenotype | Association, significance  (associated allele/genotype/ haplotype) | Reference |
| --- | --- | --- | --- | --- | --- |
| 1 | rs2820436 | *LYPLALI* | HОА | OR=0.93, р=9.4x10^-09^ (C) | Styrkarsdottir et al., 2018 |
|  |  |  | ОА | OR=0.93, р=2.01x10^-09^ (C) | Zengini et al., 2018 |
|  |  |  | КОА and/or HОА | OR=0.95, р=2.44x10^-09^ (C) | Tachmazidou et al., 2019 |
| 1 | rs2820443 | *LYPLALI* | КОА and/or HОА | OR=1.06, р=6.01x10^-11^ (C) | Tachmazidou et al. 2019 |
| 2 | rs3771501 | *TGFA* | HОА | OR=0.93, p=1.7 x10^-08^ (G) | Styrkarsdottir et al., 2018 |
|  |  |  | ОА | OR=0.94, p=1.66 x10^-08^ (G) | Zengini et al., 2018 |
|  |  |  | КОА and/or HОА | OR=0.95, p=1.84 x10^-12^ (G) | Tachmazidou et al., 2019 |
|  |  |  | HОА | OR=0.92, p=1.05 x10^-13^ (G) | Tachmazidou et al., 2019 |
|  |  |  | OA | OR=0.96, p=4.24x10^-16^ (G) | Tachmazidou et al., 2019 |
|  |  |  | Hand ОА | OR=1.08, p=1.9 x10^–10^ (А) | Boer et al., 2021 |
|  |  |  | THR | OR=1.07, p=1.6 x10^–10^ (А) | Boer et al., 2021 |
|  |  |  | TJR | OR=1.06, p=8.3 x10^–11^ (А) | Boer et al., 2021 |
|  |  |  | КОА and/or HОА | OR=1.04, p=8.8 x10^–13^ (А) | Boer et al., 2021 |
|  |  |  | HОА | OR=1.07, p=6.6 x10^–13^ (А) | Boer et al., 2021 |
|  |  |  | OA | OR=1.04, p=4.05x10^–15^ (А) | Boer et al., 2021 |
| 12 | rs1060105 | *SBNO1* | КОА | OR=1.07, p=1.9x10^-08^ (С) | Styrkarsdottir et al., 2018 |
| 12 | rs56116847 | *SBNO1* | КОА | OR=1.06, р=3.19x10^-10^ (A) | Tachmazidou et al., 2019 |
| 16 | rs6499244 | *NFAT5* | КОА | OR=1.06, р=3.88x10^-11^ (A) | Tachmazidou et al., 2019 |
| 20 | rs34195470 | *WWP2* | КОА | OR=1.07, р=2.7x10^-11^ (G) | Styrkarsdottir et al., 2018 |
|  |  |  | TKR | OR=0.93, p=3.2x10^-10^ (А) | Boer et al., 2021 |
|  |  |  | КОА | OR=0.95, p=3.1x10^-13^ (А) | Boer et al., 2021 |
| 20 | rs143384 | *GDF5* | OA | OR=1.05, р=2.1x10^-10^(A) | Styrkarsdottir et al., 2018 |
|  |  |  | КОА | OR=1.10, р=1.4x10^-19^(A) | Styrkarsdottir et al., 2018 |
|  |  |  | КОА | OR=0.91, р=4.2x10^-23^ (G) | Styrkarsdottir et al., 2019 |
|  |  |  | КОА | OR=1.1, р=4.77x10^-23^ (A) | Tachmazidou et al., 2019 |
|  |  |  | OA | OR=1.03, p=3.04x10^–11^ (А) | Boer et al., 2021 |
|  |  |  | TJR | OR=1.06, p=5.9x10^–12^ (А) | Boer et al., 2021 |
|  |  |  | TKR | OR=1.10, p=6.2x10^–15^ (А) | Boer et al., 2021 |
|  |  |  | КОА and/or HОА | OR=1.06, p=1.2x10^–20^ (А) | Boer et al., 2021 |
|  |  |  | КОА | OR=1.07, p=1.01x10^–23^ (А) | Boer et al., 2021 |

Note: ОА–оsteoarthritis; KOA–knee osteoarthritis; HOA–hip osteoarthritis; КОА and/or HОА–knee and/or hip osteoarthritis; TKR–total knee replacement; THR–total hip replacement; TJR–total joint replacement.
