# Supplementary material for: Polymorphism rs143384 GDF5 reduces the risk of knee osteoarthritis development in obese individuals and increases the disease risk in non-obese population
Source: Arthroplasty. 2024 Mar 1;6:12. doi: 10.1186/s42836-023-00229-9 (PMC10905832; doi:10.1186/s42836-023-00229-9)
Supplement: Supplementary file 3 — Additional file 3: Table S3. Sequence of oligonucleotide primers and probes for SNP candidate genes for KОА. [file 42836_2023_229_MOESM3_ESM.docx]

Supplementary table S3

Sequence of oligonucleotide primers and probes for SNP candidate genes for КОА

| Chr | SNP | Gene | Sequence of oligonucleotide primers and probes |
| --- | --- | --- | --- |
| 1 | rs2820436 | *LYPLAL1* | F: TTCCCTAATACCTATTTCAG  R: CTTGCTCTTTTCTTCCTC  FAM-atctttcCccActTttagact-BHQ-1  VIC-atctttcCccCctTttagact-BHQ-2 |
| 1 | rs2820443 | *LYPLAL1* | F: CTCTGTTTCCAGTTATCA  R: CAAGCCAAAGTATATAAAGG  FAM-agcagtTtgTttActggc-BHQ-1  VIC-agcagtTtgCttActggc-BHQ-2 |
| 2 | rs3771501 | *TGFA* | F: CAGAGAAGGTAAAAGATAAGA  R: GTGTGTGAGTAAAAGGAA  FAM-ctgtattCtaTaaGtgaacaca-BHQ-1  VIC-ctgtattCtaCaaGtgaacaca-BHQ-2 |
| 12 | rs1060105 | *SBNO1* | F: GGCATCAGATTCACTTCC  R: GACTCTCTTGTTTGTGGC  FAM-cgtcaGaaTtgCtacca-BHQ-1  VIC-cgtcaGaaCtgCtacca-BHQ-2 |
| 12 | rs56116847 | *SBNO1* | F: AGACCCATTCATTCAGTA  R: CCACAAAGTTCATTATTTCC  FAM-ctgtTccTgtAgcct-BHQ-1  VIC-ctgtTccCgtAgcct-BHQ-2 |
| 16 | rs6499244 | *NFAT5* | F: CTAGGATTACAGGCATGA  R: GGACCTACTGAAACTATTTG  FAM-catAgCaTtAgAtCtagat-BHQ-1  VIC-catAgCaTtTgAtCtagat-BHQ-2 |
| 16 | rs34195470 | *WWP2* | F: CGTTGCAGTTTCTTTTTG  R: CCAGCTAATTTTTGTATTTTTAGTA  FAM-cagtCaaAaTaAtCtGtttc-BHQ-1  VIC-cagtCaaAaCaAtCtGtttc-BHQ-2 |
| 20 | rs143384 | *GDF5* | F: GTGAGGAGTTTGGGGAGTC  R: CTGCCGCTGCTTTTGAAA  FAM-cacCttTgcTgctgcc-BHQ-1  VIC-cacCttCgcTgctgcc-BHQ-2 |

Note: F – forward primer; R – reverse primer.
