# Supplementary material for: Polymorphism rs143384 GDF5 reduces the risk of knee osteoarthritis development in obese individuals and increases the disease risk in non-obese population
Source: Arthroplasty. 2024 Mar 1;6:12. doi: 10.1186/s42836-023-00229-9 (PMC10905832; doi:10.1186/s42836-023-00229-9)
Supplement: Supplementary file 4 — Additional file 4: Table S4. The allele and genotype frequencies of the studied SNPs in the KОА and control groups with BMI < 30. [file 42836_2023_229_MOESM4_ESM.docx]

Supplementary table S4

The allele and genotype frequencies of the studied SNPs in the КОА and control groups with BMI<30

| Chr | SNP | Gene | Minor allele | Major allele | Minor allele frequency | Number of the studied chromosomes | Genotype distribution* | H_o_ | H_e_ | Р_HWE_ |
| --- | --- | --- | --- | --- | --- | --- | --- | --- | --- | --- |
| Кnee osteoarthritis patients (n=245) | | | | | | | | | | |
| 1 | rs2820436 | *LYPLAL1* | A | C | 0.274 | 486 | 14/105/124 | 0.432 | 0.398 | 0.200 |
| 1 | rs2820443 | *LYPLAL1* | С | T | 0.251 | 490 | 16/91/138 | 0.371 | 0.376 | 0.865 |
| 2 | rs3771501 | *TGFA* | A | G | 0.431 | 490 | 50/111/84 | 0.453 | 0.490 | 0.242 |
| 12 | rs1060105 | *SBNO1* | T | C | 0.225 | 490 | 19/72/154 | 0.294 | 0.348 | 0.017 |
| 12 | rs56116847 | *SBNO1* | A | G | 0.335 | 490 | 23/118/104 | 0.482 | 0.445 | 0.251 |
| 16 | rs6499244 | *NFAT5* | A | T | 0.473 | 488 | 55/121/68 | 0.496 | 0.499 | 1.000 |
| 16 | rs34195470 | *WWP2* | A | G | 0.498 | 484 | 59/123/60 | 0.508 | 0.500 | 0.898 |
| 20 | rs143384 | *GDF5* | G | A | 0.484 | 486 | 62/111/70 | 0.457 | 0.500 | 0.199 |
| Control group (n=433) | | | | | | | | | | |
| 1 | rs2820436 | *LYPLAL1* | A | C | 0.319 | 866 | 47/182/204 | 0.420 | 0.434 | 0.507 |
| 1 | rs2820443 | *LYPLAL1* | С | T | 0.253 | 838 | 33/146/240 | 0.348 | 0.378 | 0.120 |
| 2 | rs3771501 | *TGFA* | A | G | 0.412 | 862 | 83/189/159 | 0.439 | 0.485 | 0.047 |
| 12 | rs1060105 | *SBNO1* | T | C | 0.217 | 866 | 24/140/269 | 0.323 | 0.340 | 0.322 |
| 12 | rs56116847 | *SBNO1* | A | G | 0.354 | 862 | 63/179/189 | 0.415 | 0.457 | 0.058 |
| 16 | rs6499244 | *NFAT5* | A | T | 0.467 | 866 | 102/200/131 | 0.462 | 0.498 | 0.147 |
| 16 | rs34195470 | *WWP2* | A | G | 0.482 | 864 | 101/214/117 | 0.495 | 0.499 | 0.923 |
| 20 | rs143384 | *GDF5* | G | A | 0.416 | 866 | 80/200/153 | 0.462 | 0.4858 | 0.322 |

Note: * minor allele homozygotes / heterozygotes / major allele homozygotes
