# Supplementary material for: Polymorphism rs143384 GDF5 reduces the risk of knee osteoarthritis development in obese individuals and increases the disease risk in non-obese population
Source: Arthroplasty. 2024 Mar 1;6:12. doi: 10.1186/s42836-023-00229-9 (PMC10905832; doi:10.1186/s42836-023-00229-9)
Supplement: Supplementary file 5 — Additional file 5: Table S5. The allele and genotype frequencies of the studied SNPs in the KОА and control groups with BMI ≥ 30. [file 42836_2023_229_MOESM5_ESM.docx]

Supplementary table S5

The allele and genotype frequencies of the studied SNPs in the КОА and control groups with BMI≥30

| Chr | SNP | Gene | Minor allele | Major allele | Minor allele frequency | Number of the studied chromosomes | Genotype distribution* | H_o_ | H_e_ | Р_HWE_ |
| --- | --- | --- | --- | --- | --- | --- | --- | --- | --- | --- |
| Кnee osteoarthritis patients (n=255) | | | | | | | | | | |
| 1 | rs2820436 | *LYPLAL1* | A | C | 0.292 | 510 | 24/101/130 | 0.396 | 0.413 | 0.544 |
| 1 | rs2820443 | *LYPLAL1* | С | T | 0.284 | 510 | 24/97/134 | 0.380 | 0.407 | 0.285 |
| 2 | rs3771501 | *TGFA* | A | G | 0.435 | 508 | 46/129/79 | 0.508 | 0.492 | 0.702 |
| 12 | rs1060105 | *SBNO1* | T | C | 0.214 | 510 | 11/87/157 | 0.341 | 0.336 | 1.000 |
| 12 | rs56116847 | *SBNO1* | A | G | 0.345 | 510 | 37/102/116 | 0.400 | 0.452 | 0.072 |
| 16 | rs6499244 | *NFAT5* | A | T | 0.453 | 510 | 49/133/73 | 0.522 | 0.496 | 0.449 |
| 16 | rs34195470 | *WWP2* | A | G | 0.455 | 510 | 51/130/74 | 0.510 | 0.496 | 0.706 |
| 20 | rs143384 | *GDF5* | G | A | 0.424 | 510 | 44/128/83 | 0.502 | 0.488 | 0.702 |
| Control group (n=167) | | | | | | | | | | |
| 1 | rs2820436 | *LYPLAL1* | A | C | 0.362 | 334 | 22/77/68 | 0.461 | 0.462 | 0.978 |
| 1 | rs2820443 | *LYPLAL1* | С | T | 0.308 | 334 | 21/61/85 | 0.365 | 0.427 | 0.063 |
| 2 | rs3771501 | *TGFA* | A | G | 0.467 | 334 | 37/82/48 | 0.491 | 0.498 | 0.860 |
| 12 | rs1060105 | *SBNO1* | T | C | 0.177 | 334 | 7/45/115 | 0.269 | 0.291 | 0.341 |
| 12 | rs56116847 | *SBNO1* | A | G | 0.380 | 334 | 20/87/60 | 0.521 | 0.471 | 0.174 |
| 16 | rs6499244 | *NFAT5* | A | T | 0.395 | 334 | 30/72/65 | 0.431 | 0.478 | 0.205 |
| 16 | rs34195470 | *WWP2* | A | G | 0.437 | 334 | 27/92/48 | 0.551 | 0.492 | 0.123 |
| 20 | rs143384 | *GDF5* | G | A | 0.467 | 334 | 42/72/53 | 0.431 | 0.498 | 0.083 |

Note: * minor allele homozygotes / heterozygotes / major allele homozygotes
